# ENDOSCOPIC REMOVAL OF AN UNUSUAL FOREIGN BODY FROM STOMACH: A WRISTWATCH

**DOI:** 10.1590/0102-672020180001e1445

**Published:** 2019-08-26

**Authors:** Omer Faruk OZKAN, Sukru TAS, Erdem AKBAL

**Affiliations:** 1 Canakkale 18 March University, Faculty of Medicine, Department of General Surgery;; 2 Canakkale 18 March University, Faculty of Medicine, Departmentof Gastroenterology, Canakkale, Turkey

**Keywords:** Endoscopy, Foreign Bodies, Stomach, Endoscopia, Corpos Estranhos, Estômago

## INTRODUCTION

Foreign body ingestion is an important problem in adult with psychological disorders. In literature ingestion such as fish bone, fork and several metallic elements were reported. The first attempt, after diagnosis, is endoscopic removal^1^. Surgical approach is necessary in it´s failure. In this paper is presented a successful endoscopic removal of a wristwatch which was ingested by a deaf patient.

## CASE REPORT

**FIGURE 1:**
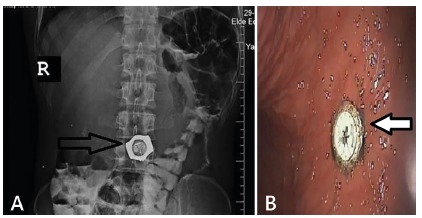
A) Abdominal plain graph was showed a watch; B) endoscopic image of the watch in the stomach

A 25-year-old male deaf patient was admitted in emergency department with complaint of abdominal pain and unable to communicate anything of his clinical history. Physical examination was normal except an epigastric tenderness. Laboratory results were normal. Direct radiography revealed a circular shaped metallic object in stomach and other several metallic objects in gastrointestinal tract (Figure 1A). An emergent gastrointestinal endoscopy was planned and during the procedure a wristwatch in the stomach was diagnosed and successfully removed with an esophageal overtube approach under direct endoscopic vision (Figure 1B).

## DISCUSSION

A delay in the diagnosis an dextraction of sharp or large sized foreign objects can lead to severe complications including mucosal laceration, obstruction, hemorrhage, and perforation. Therapeutic esophagogastroduodenoscopy with an esophageal overtube should be the first choice retrieval of large sized foreign bodies to avoid mucosal laceration, perforation and the surgical treatment. 
